# The Landcover Impact on the Aspect/Slope Accuracy Dependence of the SRTM-1 Elevation Data for the Humboldt Range

**DOI:** 10.3390/s8053134

**Published:** 2008-05-15

**Authors:** George C. Miliaresis

**Keywords:** Terrain analysis, accuracy assessment, digital elevation model, SAR

## Abstract

The U.S. National Landcover Dataset (NLCD) and the U.S National Elevation Dataset (NED) (bare earth elevations) were used in an attempt to assess to what extent the directional and slope dependency of the Shuttle Radar Topography Mission (SRTM) finished digital elevation model is affected by landcover. Four landcover classes: forest, shrubs, grass and snow cover, were included in the study area (Humboldt Range in NW portion of Nevada, USA). Statistics, rose diagrams, and frequency distributions of the elevation differences (NED-SRTM) per landcover class per geographic direction were used. The decomposition of elevation differences on the basis of aspect and slope terrain classes identifies a) over-estimation of elevation by the SRTM instrument along E, NE and N directions (negative elevation difference that decreases linearly with slope) while b) underestimation is evident towards W, SW and S directions (positive elevation difference increasing with slope). The aspect/slope/landcover elevation differences modelling overcome the systematic errors evident in the SRTM dataset and revealed vegetation height information and the snow penetration capability of the SRTM instrument. The linear regression lines per landcover class might provide means of correcting the systematic error (aspect/slope dependency) evident in SRTM dataset.

## 1. Introduction

The Shuttle Radar Topography Mission (SRTM) had successfully collected Interferometric Synthetic Aperture Radar (IFSAR) data covering over 80 percent of the landmass of the Earth by February 2000 [1]. The outcome of this effort was a digital elevation model (DEM). Several additional editing steps were applied to the SRTM DEMs [2]. The editing, also referred to as finishing, consisted of delineating and flattening water bodies, better defining coastlines, removing “spikes” and “wells”, and filling small voids. This “finished” set is publicly available at two postings: 1 arc-second for the United States and 3 arc-seconds for regions between 60 degrees N and 56 degrees S latitude. The finished SRTM data has replaced the research-grade data [3]. Research-grade SRTM data is still available from NASA's Jet Propulsion Laboratory [4].

Accuracy is computed by a comparison of DEM elevations with corresponding known elevations [5]. Test points should be well distributed, representative of the terrain, and have true elevations with accuracies well within the DEM accuracy criteria [6], [7]. Previous research efforts indicated that accuracy for a SAR derived DEM could be terrain dependent. For DEMs generated from RADARSAT, the vertical accuracy was almost linearly correlated with the terrain slope while there was no specific trend with azimuth [8]. The decomposition of SRTM 1 arc second research-grade DEM error to aspect and slope terrain classes [9] identified a) over-estimation of elevation by the SRTM instrument along certain geographic directions (negative error that decreases linearly with slope) while b) underestimation is evident towards the opposite geographic directions (positive error increasing with slope).

The SRTM radar signal measurement result in a reflective surface elevation which depends on terrain cover and is a complicated function of the electromagnetic and structural properties of the scattering medium [10]. In snow, the penetration depth of the radar signal depends on wetness, temperature, and porosity [11]. Vegetation presents an even more complex scattering environment. It has been estimated that C-band only penetrates a quarter or a third of the canopy height [12].

It is still under question if the elevation difference: bare ground elevation minus SRTM elevation data, could give an indication of the height of vegetation/buildings/snow cover. The new findings [9] that indicate the directional dependency of SRTM elevation accuracy make this task even more doubtful. The aim of the current research effort is to use landcover data [13] and the U.S National Elevation Dataset (NED) that gives bare earth elevations [14] in order to assess to what extent the directional and slope dependency [9] of the SRTM-1 finished DEM is affected by landcover.

## 2. Methodology

First the study area and the DEM data, the derivative products (slope and aspect) used as well as the landcover data are presented. The data was downloaded from the US Geological Survey data distribution system [2]. Then, the statistical analysis of the elevation difference image (NED minus SRTM) per selected landcover classes is performed. Statistics are computed for aspect and slope classes in an attempt to model the impact of direction and slope [9]. The statistical distributions were modeled on the basis of mean, standard deviation and the coefficient of skew [15].

Skewness characterizes the degree of asymmetry of a distribution around its mean [16]. The coefficient of skew is a unit-less number (Equation 1).

(1) $$\text{Skew}=\frac{n}{\left(n-1)*(n-2\right)}*\sum_{1}^{n}{\left[\frac{X_{i}-\mu }{s}\right]}^{3}$$

where n=number of points, μ= mean, s=standard deviation and Xi= the elevation difference of the i_th_ test point.

Positive skewness indicates a distribution with an asymmetric tail extending toward more positive values. Negative skewness indicates a distribution with an asymmetric tail extending toward more negative values. According to an empirical rule [6], when the absolute value of the skew exceeds a value such as 0.5, then the error distribution is sufficiently asymmetrical to cause concern that the dataset may not represent a normal distribution.

### 2.1. Study area

The study area corresponds to a NW portion of the state of Nevada (U.S.A.) that includes the Humboldt Range, with latitude in the range 40.31458° to 40.68847° (N) and longitude in the range -118.30319° to -118.00930° (W). The Humboldt Range is formed by gently sloping mountain sides while the dichotomic drainage pattern indicates that the range is surrounded by coalescent alluvial fans [9].

### 2.2 Bare earth DEM, slope and aspect

**Figure 1.**
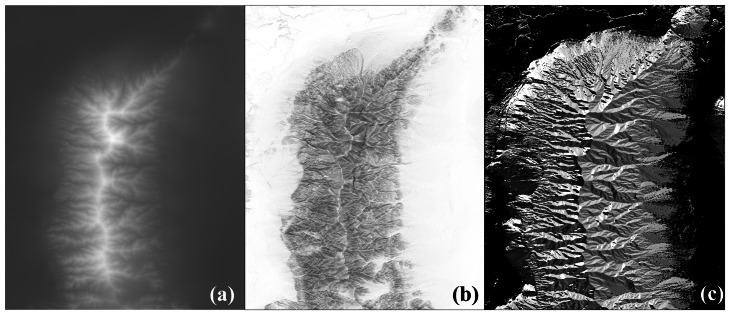
(a) Elevation is in the range 1,237 to 2,993 m, the brightest pixels have highest elevation. (b) Slope is in the range [0 to 39°], the brightest pixels present the lowest slope. (c) Aspect is quantified to the directions defined in a raster image (E, NE, N, NW, W, SW, S, SE) while the zero label was used for flat terrain (if slope is less than 1°, aspect was considered to be undefined).

NED was used as the reference DEM. NED is a digital terrain model depicting bare earth (ground) elevation in geographic co-ordinates (horizontal datum of NAD83, vertical datum of NAVD88) with spacing 1 arc second, with accuracy specification of root mean square error (RMSE) equal to 7 m [12]. The NED DEM of the study area (Figure 1a) consists of 1,346 rows and 1,058 columns. Slope (Figure 1b) and aspect, the slope pointing direction (Figure 1c) were computed on the basis of NED DEM [9]. The method that derives the topographical variables from DEMs given in geographical co-ordinates was used [17].

### 2.3 SRTM finished DEM

**Figure 2.**
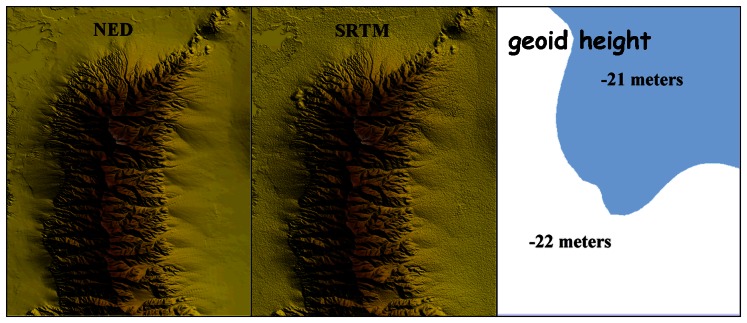
Visualization of NED and SRTM DEMs of the study area indicated the existence of random noice in SRTM elevation dataset. The geoid height in the study area (orthometric heights with respect to NAVD88 and geodetic height with respect to WGS84).

SRTM finished DEM of the study area (Figure 2a) is available [3] in geographic co-ordinates (horizontal datum of WGS84, vertical datum of EGM96) with 1 arc second spacing. The absolute horizontal and vertical accuracies are equal to 20 meters (circular error at 90% confidence) and 16 meter (linear error at 90% confidence) respectively [7]. The SRTM DEM presents an intrinsic random noise level of five m [18] that was visualized in Figure 2.

The vertical accuracy is actually significantly better than the 16 meters and it is closer to +/- 10 meters [7]. The elevations are provided with respect to the reflective surface (first return), which may be vegetation, human-made features, etc.

### 2.4 Orthometric to geodetic height recalculation

The ellipsoids (horizontal datums) GRS 80 for NAD 83 (for NED) compared to WGS 84 (for SRTM) are for all practical purposes at scales smaller than 1:5,000 identical [19], [20].

In order to conduct a realistic and consistent comparison amongst the available height data sets (vertical datum of NAVD88 for NED versus the vertical datum of EGM96 for SRTM), it was imperative that all heights refer to the same vertical datum. It was decided to perform the data comparisons in terms of ellipsoidal heights with respect to WGS84 that is consistent with the geocentric reference system employed by GPS. The difference between GPS ellipsoid height (WGS84), *h*, and levelled orthometric height, *H*, is called geoid height, *N (N*= *h*-*H)*.

Orthometric heights (NAVD88) of NED DEM were converted (recalculation of elevation values) to WGS84 ellipsoid heights [21]. The geoid height within the study area varies from -22 to -21 m (Figure 2).

The SRTM DEM grid values are provided to users in terms of orthometric heights with respect to EGM96 [22]. Ellipsoidal heights with reference to the WGS84 ellipsoid [23] were desirable. The geoidal undulations were interpolated from the EGM96 height file [24]. The geoid heights within the study area vary from -22 to -20.9 m.

**Figure 3.**
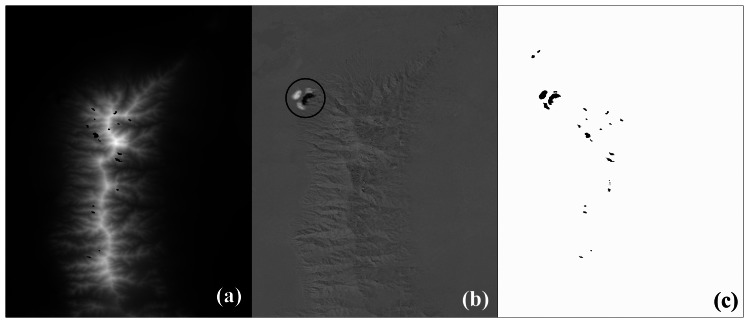
(a) SRTM-1 finished DEM. The elevation is in the range 1,235.12 to 2,989 m, the brightest pixels have highest elevation. Voids are labeled black. (b)The elevation differences (NED – SRTM) are in the range -146.3 to 128.2 m. Notice the error pattern within the circle. (c) Black points correspond either to voids or to DEM points with elevation difference not in the range [-50, 50].

**Figure 4.**
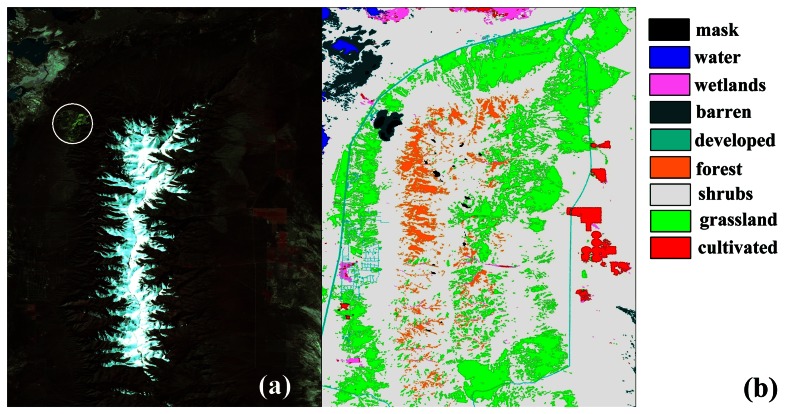
(a) The Landsat image with bands 4 (near-infrared), 3 (red), and 2 (green), displayed as red, green, and blue, respectively.A surface mine is enclosed within the circle. (b) The landcover map.

The elevation difference (NED-SRTM) per grid point was computed (Figure 3b). The visual interpretation indicated that the difference image was correlated to the aspect image (Figures 1c). An error pattern composed from dark and white regions was revealed in Figure 3b. Landsat image (Figure 4a) indicated a surface mine and a new void mask (Figure 3c) was computed.

### 2.5 Landcover

**Figure 5.**
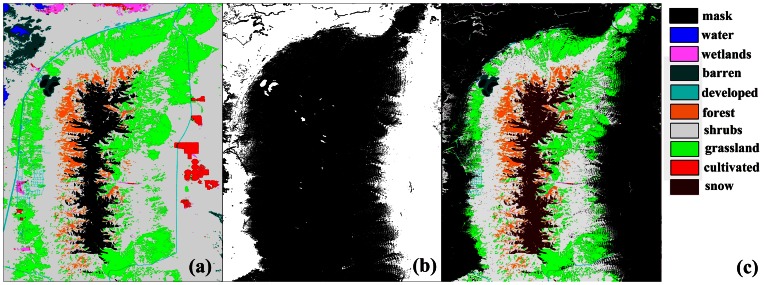
(a) A snow mask applied to the landcover map. (b) Dark points present slope greater than 2° (slope mask). (C) Landcover map that includes a snow class and the slope mask.

The National Landcover Dataset (NLCD) 2001 is a Landsat based landcover database containing 21 classes of land-cover data [13]. The snow cover was interpreted from the Landsat image (Figure 3a) downloaded from the US Geological Survey data distribution system [2]. The landcover map of the study area (Figure 4b) includes eight classes (Table 1). A snow mask was applied on the landcover map of the study area (Figure 5a).

Aspect is undefined when slope is minimised [8] so a mask was created (Figure 5b) for a slope threshold equal to 2°. The slope mask was applied to the landcover map. The final landcover map that includes a snow class is given in Figure 5c. The snow cover class consists of grid points that were initially classified as forest (4.4%), shrubs (87.3%), or grass (8.2%). The occurrence (percent area extent) in the final landcover map of forest, shrubs, grass and snow cover is 5.3%, 56.9%, 23.2%, 13% respectively (the 98.5% of the non-masked grid points).

### 2.6 Directional dependency of elevation differences

**Figure 6.**
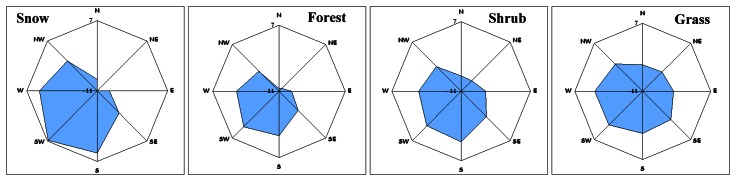
The mean elevation difference value per aspect direction per landcover class. The mean elevation difference corresponded to the radius of each rose-diagram was within the range [-11, 7] m.

Statistics and rose diagrams per landcover class per aspect direction were used in an attempt to reveal the directional dependency of elevation differences (Figure 6).

Forest and snow presented an almost similar pattern of directional dependency. SRTM instrument seems to over-estimate elevation towards the N, NE, E directions and under-estimates it towards the W, SW, S. An analogous pattern was observed for shrub and grass but the relative elevation differences were less than those observed for forest and snow (Table 2). Table 2 indicated that the magnitude of the overall mean elevation difference per landcover class was a function of mean vegetation height as it was interpreted from Table 1. That is why elevation differences were minimized for grass (Table 2).

**Figure 7.**
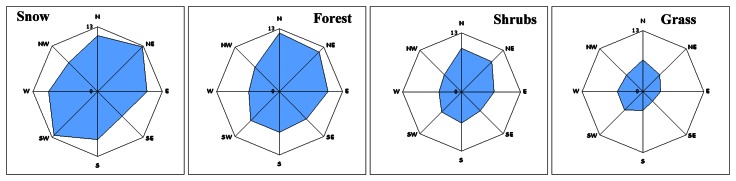
The RMSE per aspect direction per landcover class. The RMSE corresponded to the radius of each rose-diagram was within the range [0, 13] m.

RMSE is maximised for NE-SE direction for snow (Figure 7, Table 2). Forest, shrubs and grass presented an almost similar directional pattern (RMSE was maximised toward the North direction). RMSE magnitude (Table 2) seemed to be landcover dependent and interpreted to be associated to mean vegetation height estimated from Table 1. RMSE was minimised for the grass class.

**Figure 8.**
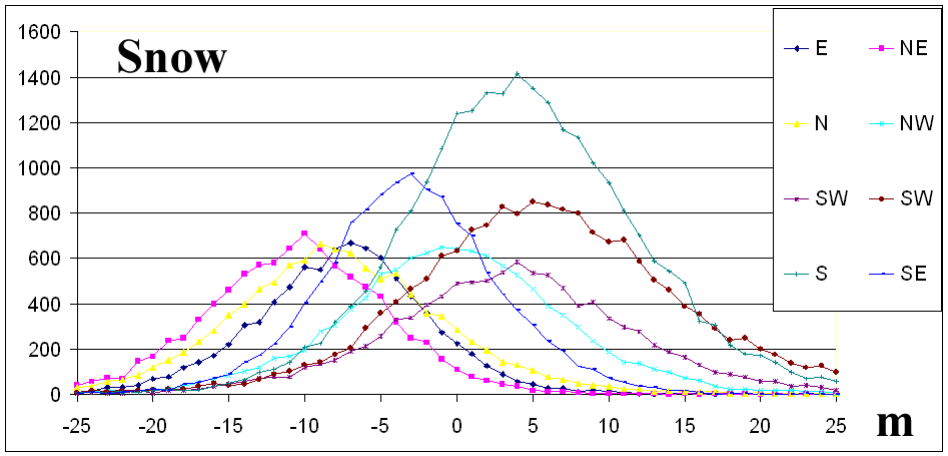
The frequency distributions per geographic direction for snow. The y-axis represents number of grid points per 1 m elevation difference.

**Figure 9.**
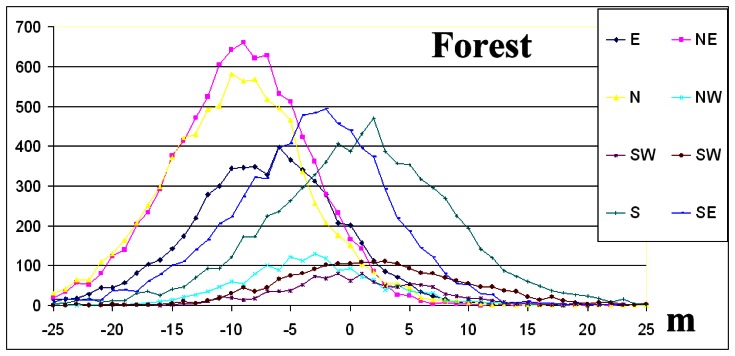
The frequency distributions per geographic direction for forest. The y-axis represents number of grid points per 1 m elevation difference.

The elevation difference frequency distributions per aspect direction for forest and snow (Figures 8, 9) as well as the statistical data of Table 2, indicate that the absolute value of the skew is less than 0.5 and normal distribution criterion is fulfilled [5].

**Figure 10.**
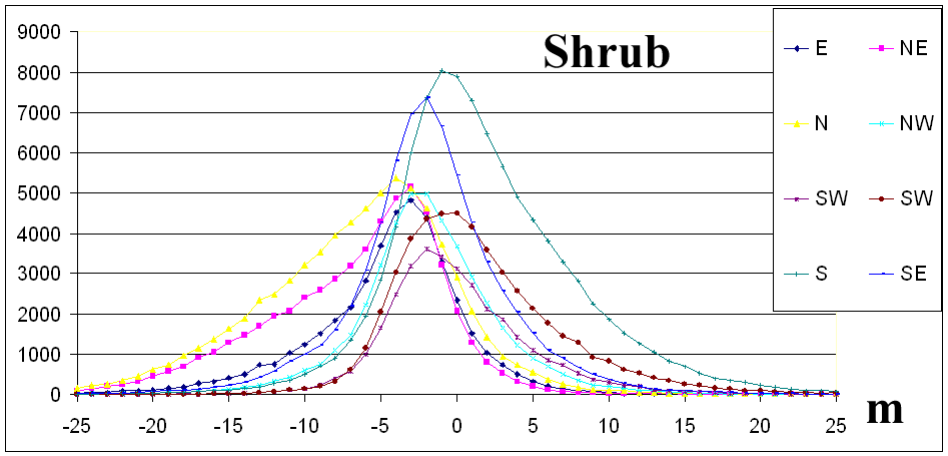
The frequency distributions per geographic direction for shrub. The y-axis represents number of grid points per 1 m elevation difference.

**Figure 11.**
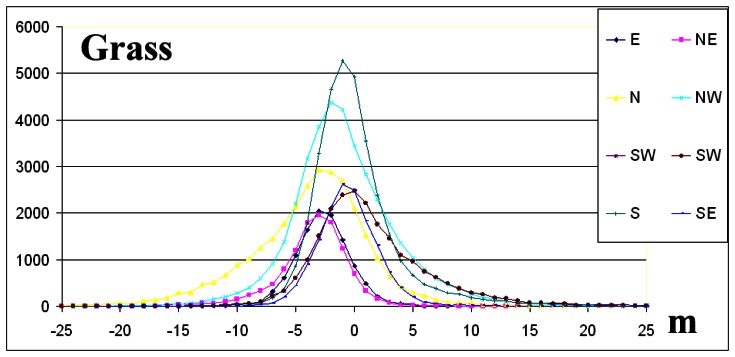
The frequency distributions per geographic direction for grass. The y-axis represents number of grid points per 1 m elevation difference.

On the contrary, for grass and shrub landcover classes (Figures 10, 11) and for the majority of geographic directions, the absolute value of the skew exceeds 0.5 (Table 2), and so the distributions are sufficiently asymmetrical to cause concern that the dataset may not represent a normal distribution [5]. The interpretation given is that although these landcover classes were dominated by shrubs or grass, trees or man-made elevated features of greater height exist (Table 1).

The interpretation of frequency distributions (Figures 8, 9 and 10) per landcover class (the grass landcover class was excluded since the directional dependency of elevation differences was minimised) revealed that elevation difference for grid points that slopes in opposite geographic directions (Table 2) was maximized, an exception being the NW-SE direction.

A two-sample means test (Equation 2) is applied [14], the null hypotheses being that for NE and SW directions of the forest class, the mean elevation difference is statistically the same.

(2) $$t=\frac{X-Y}{\sqrt{\frac{Sx^{2}}{nx-1}+\frac{Sy^{2}}{ny-1}}}$$

where X and Y correspond to the means of the two populations compared, Sx and Sy, the corresponding standard deviations, nx and ny the sample size.

The mean elevation difference was proved statistically significant since the observed t-statistics equal to 61.349 that was far greater than the tabled critical value (2.326) of t (one-tailed test, for infinite degrees of freedom at the 0.01 level). So the null hypothesis was rejected.

### 2.7 Slope dependency of elevation differences

**Figure 12.**
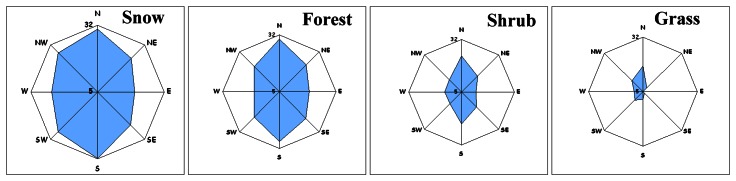
The mean slope per aspect direction per landcover class. The slope corresponded to the radius of each rose-diagram is within the range [4.7°, 32°].

Mean slope per geographic direction per landcover class is presented in Table 3, expressed as well as rose-diagrams (Figure 12).

**Figure 13.**
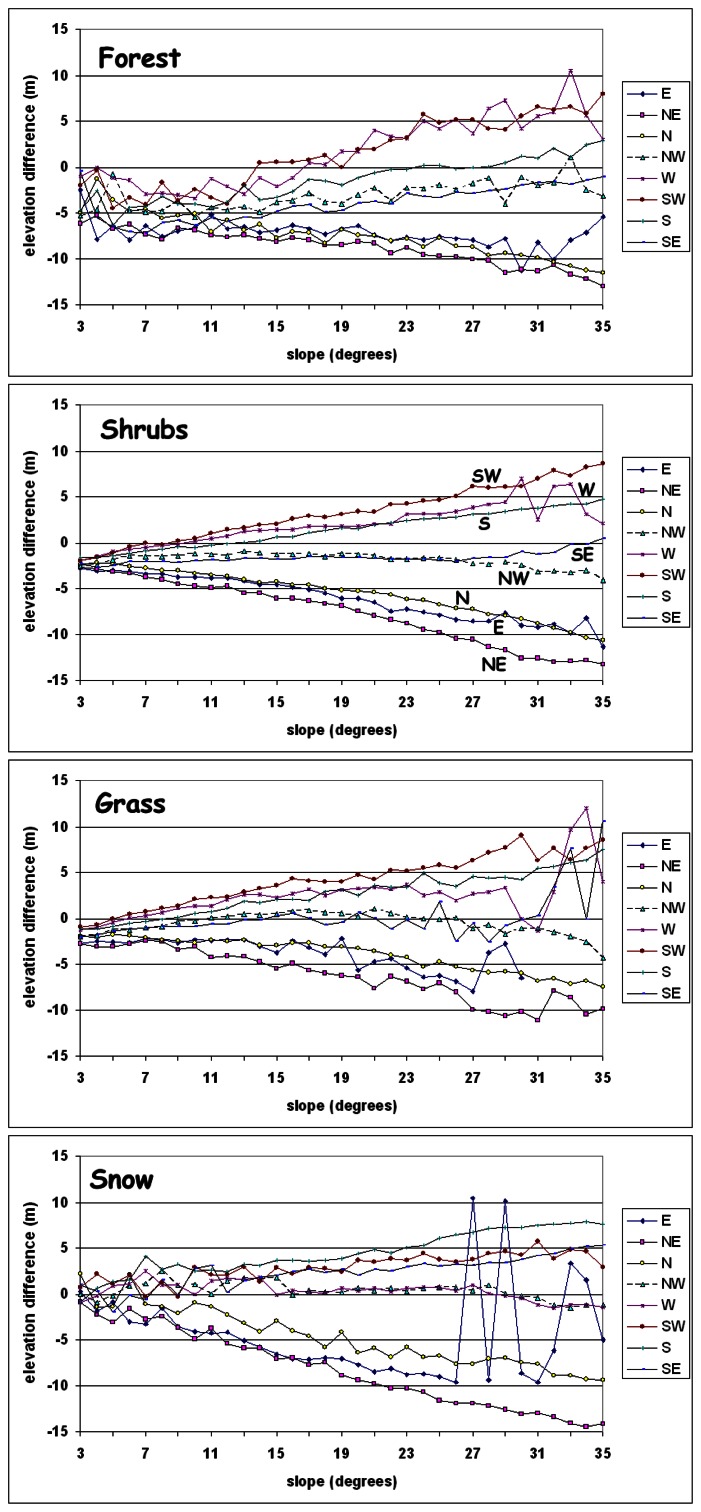
Mean elevation difference (y) per 1° slope classes per landcover class.

The relationship between one-degree slope intervals (x) and the corresponding mean elevation difference (y) is further explored by assuming the linear regression model (Figure 13, Tables 4). Y′ corresponds to the estimated value, (y′=a*x+b).

Elevation differences are linearly correlated to the terrain slope (the steeper the slope, the greater the |error|) for the eight principal geographic directions. The eight lines that correspond to the eight geographic directions per landcover class were interpreted to intersect Y-axis at a common point that corresponds (y-coordinate) to the mean vegetation height (derived from Table 1).

For snow landcover class this point approach zero (C-band was proved to penetrate snow). The lines for the forest class were interpreted to be quite noisy (due to the complex interaction of tree canopy to the radar signal) than the corresponding lines of the shrub class (Figure 13).

## 3. Discussion

The SRTM radar signal measurement results in a reflective surface elevation which depends on terrain cover. The degree of penetration depends on vegetation gap structure, canopy structure (multiple or single canopy), leaf-on versus leaf-off, wetness, ground reflectivity, and tree type [10]. The penetration depth of the radar signal depends on wetness, temperature, and porosity of snow cover [11]. These properties certainly are not constant and depend on many factors (surface, elevation, month, climatic zone, etc.). The landcover classes (Table 1) consisted of a mixture of landcover types with a specific type to prevail. Additionally the NLCD 2001 landcover database was assumed to coincide to the landcover evident during the time of SRTM data acquisition. NED DEM accounts for bare earth elevation. Both data sources are not perfect and error is evident. According to RMSE values, NED DEM is of greatest accuracy (RMSE<7 m) than SRTM DEM (RMSE<10 m) and thus it can be used for SRTM evaluation purposes [4].

The interpretation of frequency distributions (Figures 8, 9, 10 and 11) per landcover class revealed that elevation differences for grid points that slope in opposite geographic directions were maximized, an exception being the NW-SE direction (Table 2).

Figure 12, proved that in the particular physiographic region under study, the slope was maximized along the N to S geographic direction. Snow, forest and shrub presented similar directional pattern of mean slope that differed only in slope magnitude. Grass is developed over a terrain class where slope is minimized. The visual comparison of the slope direction pattern (Figure 12) to the directional patterns of mean elevation difference (Figure 6) and RMSE (Figure 7) proved that they differ.

The decomposition of elevation differences on the basis of aspect and slope terrain classes (Figure 13, Tables 4) identified: a) an over-estimation of elevation by the SRTM instrument along E, NE and N directions (negative elevation difference that decreases linearly with slope) and b) an under-estimation was evident towards W, SW and S directions (positive elevation difference increasing with slope). The elevation differences were minimised and appeared to be independent of slope magnitude along the NW and SE directions.

Elevation underestimation is a key issue, and the factors associated with it are still unexplored [8] although it is definitely geographic direction dependent as it was also proved for both the 1 and the 3 arc seconds SRTM dataset [9, 16]. Mis-registration on SRTM and reference DEM might lead to correlation between elevation differences and aspect [26], but such an assumption would be valid if tested with DEMs that were not derived from SAR imagery.

The study of three different landcover types (forest, shrub and grass with expected mean vegetation height greater than 5 m, less than 5 m and less than 0.5 m, respectively) indicated that elevation differences were mean vegetation height dependent (Figure 13). On the contrary, the SRTM (C band) signal was interpreted (Figure 13) to penetrate snow cover [10].

## 4. Conclusions

The decomposition of elevation differences on the basis of aspect and slope terrain classes identifies a) over-estimation of elevation by the SRTM instrument along E, NE and N directions (negative elevation difference that decreases linearly with slope) while b) under-estimation is evident towards W, SW and S directions (positive elevation difference increasing with slope). The aspect/slope/landcover elevation differences modelling overcome the systematic errors evident in the SRTM dataset and revealed vegetation height information and the snow penetration capability of the SRTM instrument. The linear regression lines per landcover class might provide means of correcting the systematic error (aspect/slope dependency) evident in SRTM dataset.

## Figures and Tables

**Table 1.** The included classes of the landcover map (Figure 3b).

**ID**	**Class**	**Description**
1	Water	All areas of open water, generally with less than 25% cover or vegetation or soil
2	Developed	Includes areas with a mixture of constructed materials and vegetation. These areas most commonly include single-family housing units.
3	Barren	Barren areas of bedrock. Vegetation accounts for less than 15% of total cover.
4	Forest	Areas dominated by trees generally greater than 5 meters tall, and greater than 20% of total vegetation cover.
5	Shrub	Areas dominated by shrubs; less than 5 meters tall with shrub canopy, typically greater than 20% of total vegetation.
6	Grass	Areas dominated by grammanoid vegetation, generally greater than 80% of total vegetation.
7	Cultivated	Crop vegetation accounts for greater than 20 percent of total vegetation. Forest or shrub land vegetation or perennial herbaceous vegetation
8	Wetlands	accounts for greater than 20 percent of vegetative cover and the soil or substrate is periodically saturated or covered with water.

**Table 2.** For a certain landcover class, the number of points, the mean, the standard deviation, the coefficient of skew and RMSE of the elevation differences are presented per aspect direction.

**Class**	**Attribute**	**Aspect direction**								
		**E**	**NE**	**N**	**NW**	**W**	**SW**	**S**	**SE**	**All**
**Forest**	Points	5726	9,130	8424	1611	1046	2006	7518	7382	42843
	Mean	-7.2	-9.4	-9.7	-3.2	0.4	2.3	0.9	-3.5	-5.3
	St.dev.	6.4	5.9	6.4	6.0	6.1	7.8	7.9	6.8	8.0
	Skew	-0.2	-0.3	0.0	0.2	0.0	0.4	-0.2	-0.4	0.2
	RMSE	9.6	11.1	11.7	6.8	6.1	8.1	8.0	7.7	9.6

**Grass**	Points	11099	11842	29463	38139	22258	22258	33130	15121	168189
	Mean	-2.7	-3.6	-3.8	-0.8	1.5	1.5	0.1	-0.5	-0.9
	St.dev.	2.5	3.3	5.3	4.7	5.1	5.1	3.8	2.7	5.0
	Skew	0.0	-0.9	-0.2	0.5	0.9	0.8	0.9	0.8	0.6
	RMSE	3.6	4.9	6.5	4.8	5.3	5.3	3.8	2.8	5.1

**Shrub**	Points	42327	55901	71253	44784	33061	50056	93974	67236	458592
	Mean	-4.5	-6.7	-6.7	-1.9	-0.1	1.4	1.9	-1.8	-2.2
	St.dev.	5.2	6.0	6.5	4.8	4.8	5.7	6.3	5.3	6.7
	Skew	-0.4	-0.6	-0.3	0.1	0.6	0.9	0.7	0.2	0.0
	RMSE	6.9	9.1	9.3	5.2	4.8	5.9	6.5	5.6	7.0

**Snow**	Points	8555	9381	10419	11236	10250	17632	24907	12836	105216
	Mean	-7.5	-10.5	-7.7	-0.3	3.5	6.3	4.5	-3.1	-0.3
	St.dev.	5.9	6.3	7.5	7.9	8.8	10.0	8.1	6.0	9.9
	Skew	0.1	0.4	0.4	0.1	0.0	0.2	0.3	0.1	0.3
	RMSE	9.5	12.3	10.8	7.9	9.5	11.9	9.2	6.7	9.9

**Table 3.** Mean and st.dev. of slope per geographic direction per landcover class.

**Class**	**Attribute**	**Slope in degrees**									
		**E**	**NE**	**N**	**NW**	**W**	**SW**	**S**	**SE**	**All**
**Forest**	Mean	18.8	22.8	29.6	21.6	16.8	21.6	28.2	22.5	24.2
	St.dev.	6.1	7.2	8.2	7.9	8.3	9.1	9.4	7.4	8.8

**Grass**	Mean	4.7	7.4	17.4	12.3	9.0	10.2	8.4	5.5	10.4
	St.dev.	3.4	7.1	11.4	9.2	7.4	8.6	8.2	4.4	9.3

**Shrub**	Mean	12.0	16.2	23.1	13.7	13.3	13.1	20.8	15.4	16.9
	St.dev.	8.1	10.2	15.5	9.9	16.5	9.5	12.4	10.5	11.8

**Snow**	Mean	19.7	23.8	30.1	27.1	23.3	27.2	31.4	23.4	26.7
	St.dev.	5.1	6.6	8.5	7.5	6.7	7.1	7.4	6.4	8.0

**Table 4.** Linear regression (y=ax+b) and the correlation coefficient (R) per landcover classes.

Attributes				**E**	**NE**	**N**	**NW**	**W**	**SW**	**S**	**SE**
**Forest**	b			-4.03	-5.19	-3.12	-4.59	-4.19	-5.49	-6.02	-6.94
	a			-0.20	-0.19	-0.23	0.06	0.30	0.37	0.24	0.16
a in degrees				-11.5	-10.7	-12.8	3.5	16.6	20.2	13.5	8.9
R				0.55	0.96	0.96	0.42	0.60	0.94	0.96	0.81

**Grass**		b		-1.31	-0.87	-0.50	0.44	0.56	-0.70	-1.94	-2.50
		a		-0.15	-0.29	-0.18	-0.06	0.08	0.25	0.24	0.14
a in degrees				-8.6	-16.2	-10.3	-3.5	4.3	14.1	13.7	7.8
R				0.73	0.95	0.96	0.46	0.28	0.96	0.99	0.55

**Shrubs**		b		-2.34	-1.03	-0.66	-0.86	-1.20	-2.68	-2.38	-2.83
		a		-0.18	-0.35	-0.26	-0.06	0.15	0.32	0.20	0.07
a in degrees				-10.3	-19.4	-14.5	-3.3	8.7	17.5	11.4	3.8
R				0.82	0.99	0.99	0.71	0.71	0.99	1.00	0.82

**Snow**			B	-4.06	-0.23	0.98	1.28	1.71	1.09	0.21	-0.54
			a	-0.02	-0.42	-0.29	-0.05	-0.08	0.09	0.22	0.15
a in degrees				-1.1	-22.9	-16.4	-3.1	-4.5	5.0	12.7	8.8
R				0.04	0.99	0.95	0.51	0.70	0.61	0.97	0.91
